# MicroRNA-101 inhibits cell progression and increases paclitaxel sensitivity by suppressing MCL-1 expression in human triple-negative breast cancer

**DOI:** 10.18632/oncotarget.4039

**Published:** 2015-05-08

**Authors:** Xiaoping Liu, Hailin Tang, Jianping Chen, Cailu Song, Lu Yang, Peng Liu, Neng Wang, Xinhua Xie, Xiaoti Lin, Xiaoming Xie

**Affiliations:** ^1^ Department of Breast Oncology, Sun Yat-Sen University Cancer Center, State Key Laboratory of Oncology in South China, Collaborative Innovation Center for Cancer Medicine, Guangzhou, Guangdong, China; ^2^ School of Chinese Medicine, The University of Hong Kong, Hong Kong

**Keywords:** miR-101, triple-negative breast cancer, paclitaxel, sensitivity, MCL-1

## Abstract

Triple-negative breast cancer is the most aggressive breast cancer subtype. The aim of our study was to investigate the functional role of both miR-101 and MCL-1 in the sensitivity of human triple-negative breast cancer (TNBC) to paclitaxel. We found that the expression of miR-101 was strongly decreased in triple-negative breast cancer tissues and cell lines. The expression of miR-101 was not associated with clinical stage or lymph node infiltration in TNBC. Ectopic overexpression of miR-101 inhibit growth and induced apoptosis *in vitro* and suppressed tumorigenicity *in vivo*. MCL-1 was significantly overexpressed in most of the TNBC tissues and cell lines. Luciferase assay results confirmed MCL-1 as a direct target gene of miR-101. MiR-101 inhibited MCL-1 expression in TNBC cells and transplanted tumors. There was a negative correlation between the level of expression of miR-101 and MCL-1 in TNBC tissues. Suppression of MCL-1 enhanced the sensitivity of MDA-MB-435 cells to paclitaxel. Furthermore, miR-101 increased paclitaxel sensitivity by inhibiting MCL-1 expression. Our findings provide significant insight into the molecular mechanisms of TNBC carcinogenesis and may have clinical relevance for the development of novel, targeted therapies for TNBC.

## INTRODUCTION

Breast cancer, the most common malignancy in women, is a heterogeneous disease with substantial diversity in histological and molecular characteristics that require specialized therapeutic interventions [1]. Most cases are classified as ‘sporadic’ breast carcinoma caused by genetic changes that occur over time [2], and only a small percentage (5-10%) of cases are of hereditary origin. The estrogen receptor (ER), the progesterone receptor (PR) and human epidermal growth factor receptor 2 (HER2) are the molecular biomarkers currently used in routine clinical practice to help make treatment decisions for breast cancer [3]. Triple-negative breast cancer (TNBC), which was named for the absence of ER, PR, and HER2 expression, is the most aggressive breast cancer subtype. TNBC has a high propensity for metastasis and a poor prognosis [4]. Current treatment modalities for TNBC are limited to surgery, radiation, and systemic chemotherapy due to the lack of more specific therapeutic targets. However, patients often experience early relapse from distant tumor metastasis even if they initially respond well to the treatments. Over the past few decades, tremendous effort has been expended in the search for a molecular targeted therapy for TNBC with limited success [5].

MicroRNAs (miRNAs) are small, non-coding, endogenous RNA molecules involved in gene regulation that are located in introns of protein-coding genes, introns of non-coding genes, or exons of non-coding genes. These small (18–25 nucleotides long) non-coding RNAs function as post-transcriptional regulators of gene expression by binding to the 3′ untranslated region (UTR) of target mRNAs and promoting mRNA degradation or translational repression [6]. Several studies demonstrated that miRNAs can inhibit the expression of oncogenes and tumor suppressors [7].

Furthermore, several miRNAs that play a crucial role in TNBC biology have been identified, and these miRNAs may have therapeutic implications. The causal involvement of microRNAs in breast cancer and the possible use of these small non-coding RNA molecules as biomarkers have been extensively studied, and those studies have generated promising results. Utilizing miRNAs as a therapeutic tool might be an innovative treatment approach, especially for tumor subgroups, such as TNBC, which cannot be treated with efficient and specific therapies [8]. In breast cancer, miRNA expression profiling using microarray technology, combined with some histopathological features, such as ER, PR and HER2 status, has been established as a useful tool for classifying tumors [9]. MicroRNA-101 (miR-101) expression is negatively associated with tumor growth and blood vessel formation in several solid epithelial cancers, including breast cancer. Researchers have confirmed that miR-101 acts as a tumor suppressor in gliomas [10]. The expression of miR-101 was decreased in human breast cancer tissues and inhibited cellular proliferation as well as invasiveness by targeting Stmn1 [11]. MAGI-2 suppression by miR-101 reduces PTEN activity, leading to Akt activation in MCF-7 breast cancer cells [12]. However, the role of miR-101 in TNBC remains elusive.

Myeloid cell leukemia 1 (MCL-1), a pro-survival member of the Bcl-2 (B-cell CLL/lymphoma 2) family, has been associated with the aberrant expression of pro-survival Bcl-2 family proteins and tumorigenesis as well as resistance to chemotherapies [13]. Previous studies have demonstrated that several miRNAs induce apoptosis by targeting MCL-1 in nasopharyngeal carcinoma [14], acute myeloid leukemia [15] and ovarian cancer [16]. Our previous study showed that MCL-1 could be targeted with miR-26a in breast cancer [17]. Although studies have shown that MCL-1 is a target of miR-101 in lung cancer [18] and endometrial cancer cells [19], the roles of MCL-1 and miR-101 in drug sensitivity have not been identified.

The goal of the present study was to identify “druggable” targets for TNBC to develop new treatment options. In this study, we analyzed the expression of miR-101 by using a triple-negative breast cancer tissue microarray. Previous investigations led us to study MCL-1, a putative target of miR-101. After demonstrating the mechanism of miR-101 in *in vitro* and *in vivo* models, we hypothesized that miR-101 could be involved in the pathogenesis of breast cancer. In this study, miR-101 was considered a potential therapeutic target for TNBC.

## RESULTS

### Expression of miR-101 is decreased in TNBC cell lines and tissues

**Figure 1:**
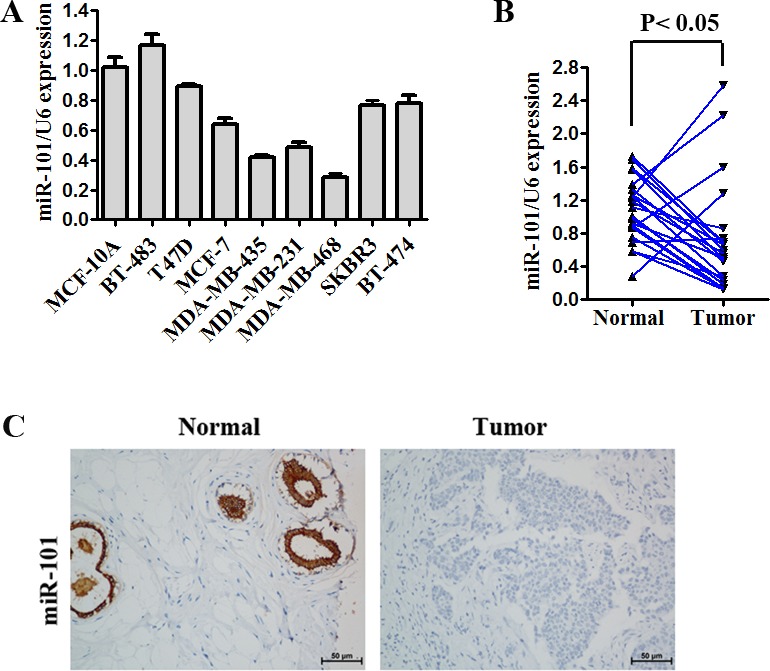
Expression of miR-101 is decreased in TNBC **A.** miR-101 expression was determined by quantitative RT-PCR in one normal mammary cell line, five tumor cell lines with a luminal transcriptional profile and three tumor cell lines with a basal-like transcriptional profile. miR-101 expression was normalized using U6 RNA expression. Error bars represent standard deviations (SD) for three replicates in one experiment. **B.** miR-101 expression in 22 paired TNBC specimens and the corresponding paired normal adjacent tissues are shown in a column analysis. **C.** Representative images of miR-101 expression as determined by ISH.

The miR-101 expression was decreased in seven of the eight breast cancer cell lines (BT-483, T47D, MCF-7, SKBR3, BT-474, MDA-MB-435, MDA-MB-231, and MDA-MB-468) compared to the normal human mammary epithelial cell line MCF-10A (Figure 1A). These results indicated that miR-101 had lower expression in basal-like cell lines compared to luminal cell lines. qRT-PCR revealed a decrease in miR-101 expression in 16 of 22 TNBC samples (72.7%) (Figure 1B). Then, using ISH, we found that miR-101 expression was decreased in 56 of 86 samples (65.1%) (Figure 1C). No significant correlations between miR-101 expression and age or tumor size were found (Table 2).

**Table 1:** Clinicopathologic characteristics of patients

Characteristics	Cohort 1	Cohort 2
	TNBC (*n* = 22)	TNBC (*n* = 86)
Age (mean ± standard deviation)	47.8 ± 14.5	48.5 ± 11.2
Tumor size (cm)	6.8 ± 1.4	6.3 ± 1.1
Stage		
I-II	15	61
III-IV	7	25
Lymph node		
Not infiltrated	10	41
Infiltrated	12	45

**Table 2:** Analysis of the correlation between miR-101 expression and clinicopathological parameters in breast cancer

Viable	Cases	miR-101		
		low	high	*p* value
Age (years) < 50 ≥ 50	43 43	28 28	15 15	0.589
Tumor size (cm) ≤ 2 > 2	23 63	14 42	9 21	0.400
Stage I-II III-IV	61 25	34 22	27 3	0.003
Lymph node Not infiltrated Infiltrated	41 45	19 37	22 18	0.032

### MiR-101 suppresses the proliferation, apoptosis and tumorigenic capacity of TNBC cells

**Figure 2:**
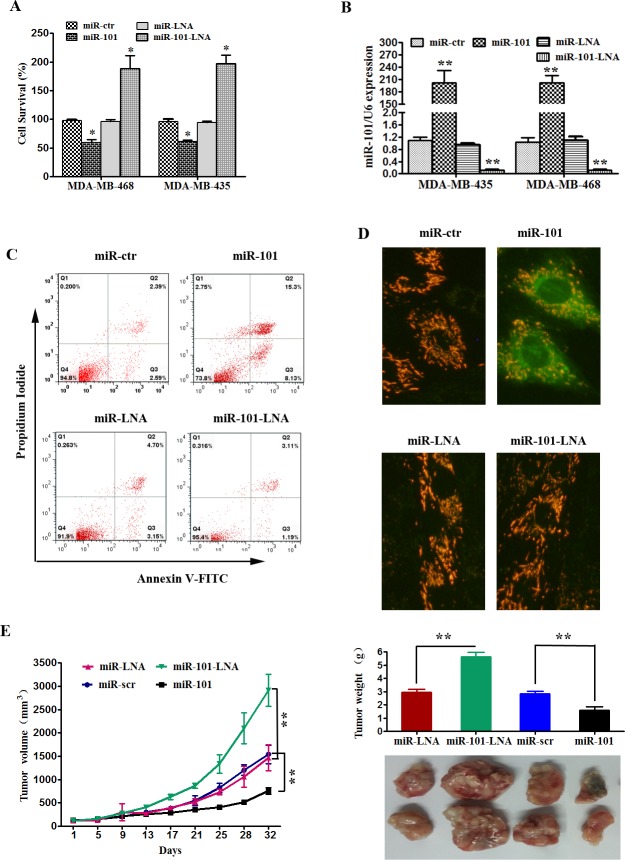
MiR-101 suppresses proliferation and induces apoptosis of TNBC **A.** MDA-MB-435 and MDA-MB-468 cell lines were transfected with miR-101 mimics, miR-101 inhibitors or their controls. Cell viability was determined using the MTT assay at 48 h post-transfection. *p < 0.05, **p < 0.01. The data represent the mean ± SD of three independent experiments. **B.** MDA-MB-435 and MDA-MB-468 cells were transfected with miR-101 mimics or scramble mimics (upper panel). The effect of transfection on the levels of miR-101 expression was determined by qRT-PCR. **p < 0.01 vs. control. **C.** Apoptosis was evaluated by analyzing Annexin V-FITC and propidium iodine staining by FACS. **D.** MDA-MB-435 cell lines were transfected with miR-101 mimics, miR-101 inhibitors, or control sequences. Mitochondrial membrane potential (MMP, Δψm) was determined at 48 h post-transfection. Green indicates apoptosis. **E.** MDA-MB-435 cells were subcutaneously injected into nude mice. Then, the effects of intratumoral injection of 40 μL of scramble, miR-101 mimic or control, or miR-101 inhibitors in PBS on tumor volume was examined. The average tumor volumes are shown (*n* = 5 for both experimental groups) starting from the first injection and continuing until after the mice were killed at day 32. After 32 days, the mice were euthanized, necropsies were performed, and the tumors were weighed. All data are shown as the mean ± SD, **p < 0.01.

To explore the functional effects of miR-101 in TNBC cells, we performed an MTT assay, flow cytometry analysis and JC-1 staining in MDA-MB-435 and MDA-MB-468 cells. As shown in Figure 2A, the rate of cell survival was considerably lower for cells transfected with miR-101 mimics compared to the respective controls. By contrast, the inhibition of miR-101 by miR-101-LNA significantly increased cell survival compared to the control (Figure 2A). To assess whether this effect is mediated through the induction of cell apoptosis, flow cytometry analysis and JC-1 staining were performed. The rate of apoptosis in MDA-MB-435 cells was increased following transfection with miR-101 mimics. Meanwhile, the inhibition of miR-101 suppressed cell apoptosis in MDA-MB-435 cells (Figure 2C). We found that the overexpression of miR-101 resulted in reduced MMP and increased green fluorescence in MDA-MB-435 cells (Figure 2D). Consistent with the *in vitro* results, the *in vivo* growth rate of MDA-MB-435 cells transfected with miR-101 mimics was significantly slower than the growth rate of the controls, while the miR-101-LNA group demonstrated a faster growth rate (Figure 2E). These observations provide strong evidence that the overexpression of miR-101 significantly suppresses the growth of TNBC cells *in vitro* and *in vivo*.

### Transfection of miR-101 sensitizes TNBC cells to paclitaxel by inducing apoptosis

**Figure 3:**
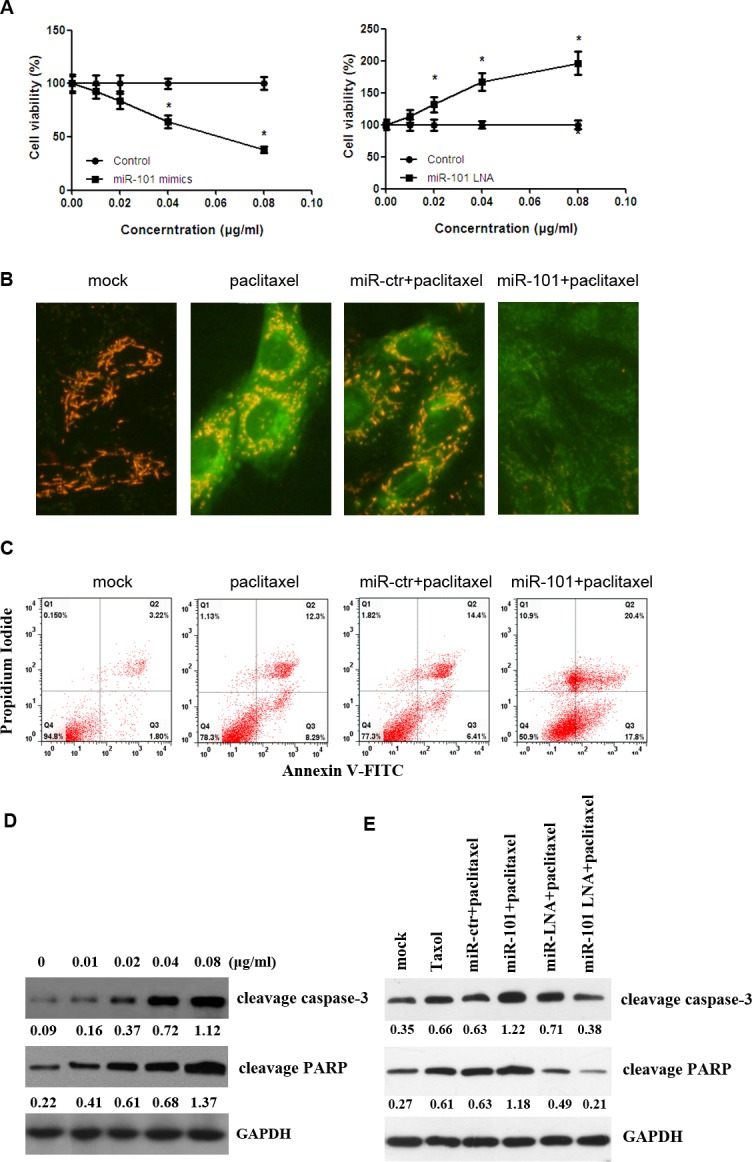
Transfection of miR-101 sensitizes breast cancer cells to paclitaxel-induced apoptosis **A.** The kinetics of the effect of miR-101 on breast cancer cell viability. MDA-MB-435 cells were transiently transfected with the indicated liposomal complexes, and the control group was measured for cell viability at 48 h post-transfection. Then, the cells were treated with different doses of paclitaxel. Data represent the mean ± SD of three independent experiments. *p < 0.05. **B.** MDA-MB-435 cells were transfected with paclitaxel, miR-101 mimics, miR-101 inhibitors or a combination. Mitochondrial membrane potential (MMP, Δψm) was determined at 48 h post-transfection. Green indicates apoptosis. **C.** MDA-MB-435 cells were transfected with paclitaxel, miR-101 mimics, miR-101 inhibitors or a combination. The apoptotic cells were evaluated with Annexin V-FITC and propidium iodine staining and then analyzed by FACS. **D.** MDA-MB-435 cells were transfected with different doses of paclitaxel. Cleaved caspase-3 and cleaved PRAP protein levels were evaluated by Western blot. **E.** MDA-MB-435 cell lines were treated with paclitaxel and transfected with miR-101 mimics, miR-101 inhibitors or a combination. Cleaved caspase-3 and cleaved PRAP protein levels were evaluated by Western blot.

In this study, ectopic overexpression of miR-101 increased paclitaxel sensitivity, whereas the inhibition of miR-101 decreased paclitaxel sensitivity in MDA-MB-435 cells (Figure 3A). Our results showed that enhanced expression of miR-101 caused a significant reduction in MMP, an increase in green fluorescence and an increase in the rate of apoptosis in MDA-MB-435 cells after paclitaxel treatment (Figure 3B and 3C). To further confirm these results, the levels of apoptotic markers were detected in transfected MDA-MB-435 cells by Western blot analysis after treatment with paclitaxel. First, we detected the effect of paclitaxel on apoptosis in MDA-MB-435 cells. Increasing doses of paclitaxel enhanced the expression of cleaved caspase-3 and PARP (Figure 3D). Western blot analysis showed that the overexpression of miR-101 significantly increased the expression of cleaved caspase-3 and PARP (Figure 3E).

### MCL-1 is a target of miR-101 in breast cancer cells

**Figure 4:**
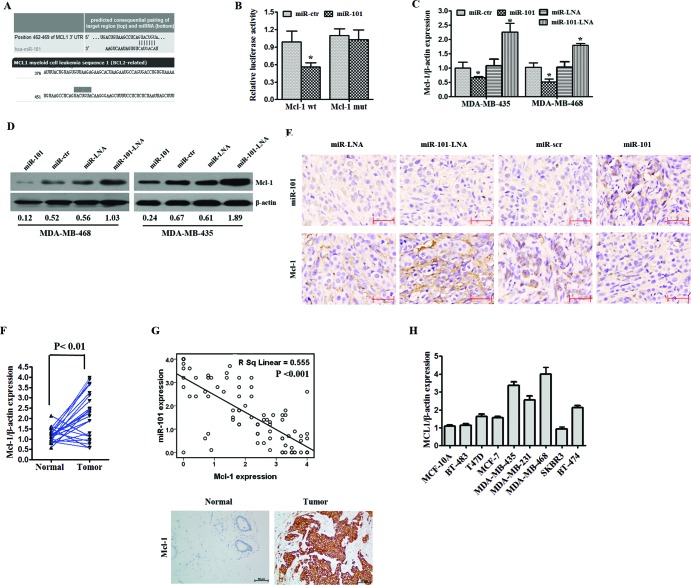
MiR-101 directly targets MCL-1 **A.** MiR-101 was predicted to bind to the MCL-1 3′-UTR using TargetScan and microRNA online software. **B.** A luciferase assay was performed on MDA-MB-435 cells co-transfected with miR-101 mimics, a scrambled control, miR-101 inhibitors, a control and a luciferase reporter containing MCL-1 3′-UTR (MCL-1-wt) or mutant constructs in which the five nucleotides of the miR-101 binding site were mutated (MCL-1-mut). An empty luciferase reporter construct was used as a negative control. *p < 0.05 vs. scramble. **C.** MDA-MB-435 and MDA-MB-468 cell lines were transfected with miR-101 mimics, miR-101 inhibitors or their controls. The mRNA expression of MCL-1 was detected by qRT-PCR. **D.** The effect of miR-101 mimics or miR-101 inhibitors on the protein expression of MCL-1 was determined by Western blot in both the MDA-MB-435 and MDA-MB-468 cell lines. β-actin was used as a loading control. **E.** In situ hybridization was used to detect the expression of miR-101, and immunohistochemistry was used to detect the expression of MCL-1 in transplanted tumor tissues in the scramble, miR-101 mimics, control and miR-101 inhibitor groups. **F.** The expression of MCL-1 in 22 paired TNBC specimens and the corresponding paired normal, adjacent tissues are shown in a column analysis. **G.** The correlation between miR-101 and MCL-1 expression in breast cancer tissues was analyzed by comparing miRNA and mRNA expression. **H.** The expression of MCL-1 was determined by qRT-PCR in one normal mammary cell line, five tumor cell lines with a luminal transcriptional profile and three tumor cell lines with a basal-like transcriptional profile. miR-101 expression was normalized using U6 RNA expression. Error bars represent standard deviations (SD) for three replicates in one experiment.

TargetScan was used to help identify miR-101 targets in human TNBC. Among the candidate target genes, the algorithm predicted MCL-1 as a target (Figure 4A). The miR-101 mimics, but not miR-ctr, specifically decreased luciferase expression of the MCL1-3′-UTR-wt reporter. By contrast, no change in relative luciferase expression was observed in cells transfected with the MCL1-3′-UTR-mut reporter (Figure 4B). These results suggest that MCL-1 is a direct target of miR-101. The results of qRT-PCR and Western blot analyses showed that enhanced expression of miR-101 by miR-101 mimics in MDA-MB-435 and MDA-MB-468 cells led to the downregulation of endogenous MCL-1 mRNA and decreased protein levels. Accordingly, the inhibition of endogenous miR-101 by a miR-101 inhibitor (miR-101-LNA) resulted in the upregulation of endogenous MCL-1 mRNA and protein levels compared to the negative control (Figure 4C and 4D). In transplanted tumors, the expression of MCL-1 was decreased in the miR-101-treated group (Figure 4E). The expression of MCL-1 mRNA was significantly increased in 77.2% (17/22) of tumor tissues (Figure 4F), and the protein level was overexpressed in all three TNBC cell lines (Figure 4H). Furthermore, miR-101 expression was negatively correlated with MCL-1 expression in TNBC tissues (R = 0.555, *P* < 0.001; Figure 4G).

### MCL-1 mediates the suppressive functions of miR-101 in TNBC cells

**Figure 5:**
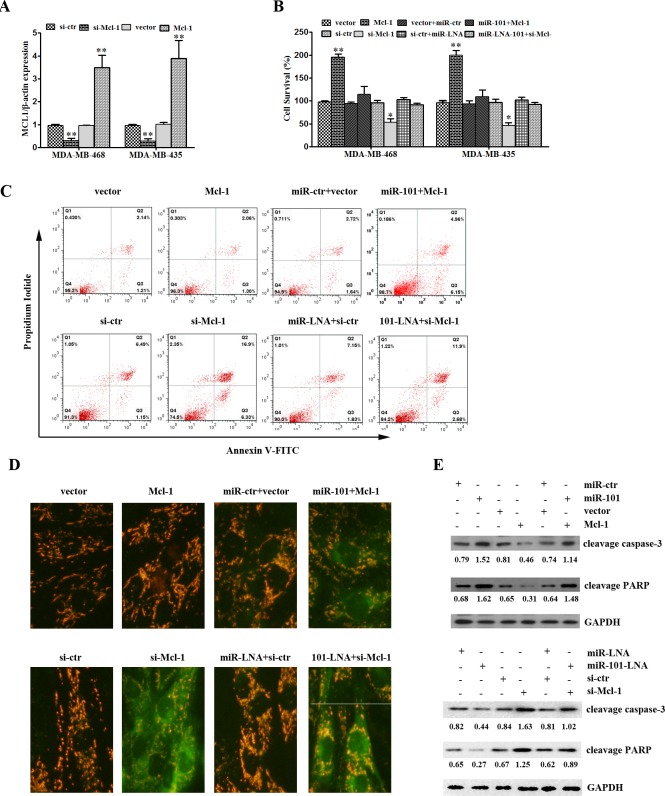
MiR-101 suppresses proliferation and induces apoptosis of TNBC by targeting MCL-1 **A.** MDA-MB-435 and MDA-MB-468 cells were transfected with an MCL-1 overexpression vector or mock vector. The effect of transfection on the levels of miR-101 expression was determined by qRT-PCR. **p < 0.01 vs. control. **B.** MDA-MB-435 cells were transfected with miR-101 mimics, miR-101 inhibitors, a MCL-1 overexpression vector, MCL-1 siRNA or a combination. The expression of MCL-1 mRNA was detected by qRT-PCR. **C.** MDA-MB-435 cells were transfected with miR-101 mimics, miR-101 inhibitors, an MCL-1 overexpression vector, MCL-1 siRNA or a combination. The apoptotic cells were evaluated by Annexin V-FITC and propidium iodine staining and then analyzed by FACS. **D.** MDA-MB-435 cells were transfected with miR-101 mimics, miR-101 inhibitors, an MCL-1 overexpression vector, MCL-1 siRNA or a combination. Mitochondrial membrane potential (MMP, Δψm) was determined at 48 h post-transfection. Green indicates apoptosis. **E.** MDA-MB-435 cells were transfected with miR-101 mimics, miR-101 inhibitors, a MCL-1 overexpression vector, MCL-1 siRNA or a combination. Cleaved caspase-3 and cleaved PRAP protein expression was evaluated by Western blot.

The rate of cell survival was considerably lower after transfection with MCL-1 siRNA compared to respective controls in MDA-MB-468 and MDA-MB-435 cells. By contrast, the upregulation of MCL-1 by an MCL-1 overexpression vector significantly increased cell survival compared to the control (Figure 5A). In MDA-MB-468 and MDA-MB-435 cells, MCL-1 overexpression could reverse the inhibition induced by miR-101. However, MCL-1 knockdown could erase the effect of the miR-101 inhibitor (Figure 5B). MCL-1 overexpression decreased the rate of apoptosis in MDA-MB-435 cells. miR-101 recovered the effect of MCL-1 suppression on apoptosis in MDA-MB-435 cells. MCL-1 knockdown increased the rate of apoptosis in MDA-MB-435 cells. miR-101 inhibition interrupted the induction of apoptosis in MDA-MB-435 cells by MCL-1 siRNA (Figure 5C). MCL-1 knockdown resulted in reduced MMP and increased green fluorescence. Additionally, the overexpression of MCL-1 could attenuate the effect of miR-101 on MMP in MDA-MB-435 cells (Figure 5D). The upregulation of MCL-1 suppressed the expression of cleaved caspase-3 and cleaved PARP, and this effect on MCL-1 was weakened by miR-101. Conversely, inhibition of MCL-1 enhanced the levels of cleaved caspase-3 and cleaved PARP, and this effect on MCL-1 was abated by miR-101 (Figure 5E).

### MCL-1 mediates miR-101-induced paclitaxel sensitivity in TNBC cells

**Figure 6:**
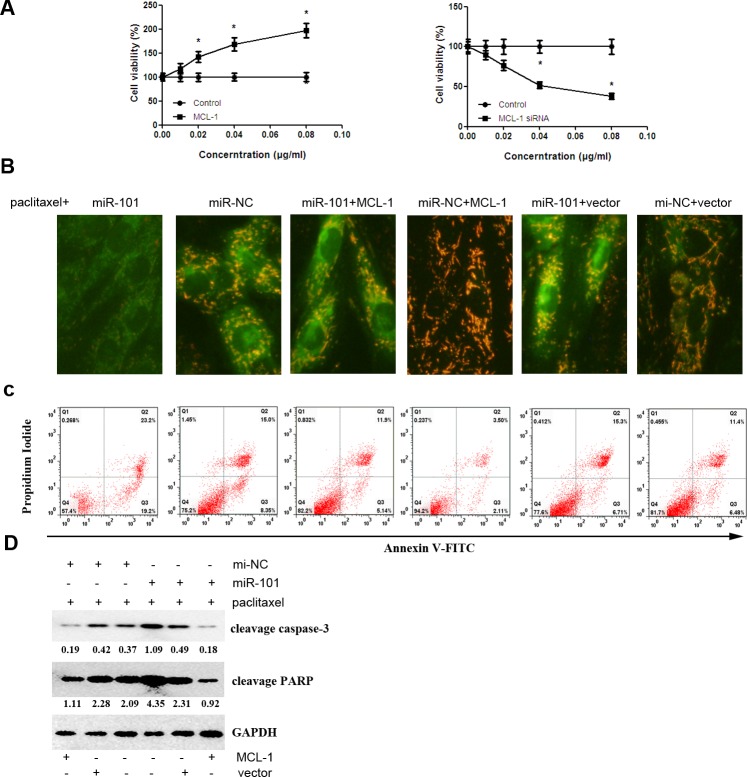
MiR-101 sensitizes TNBC cells to paclitaxel-induced apoptosis by targeting MCL-1 **A.** The kinetics of the effect of MCL-1 on breast cancer cell viability. MDA-MB-435 cells were transiently transfected with the indicated liposomal complexes, and the control group was measured for cell viability at 48 h post-transfection. Then, the cells were treated with different doses of paclitaxel. The data represent the mean ± SD of three independent experiments. *p < 0.05. **B.** MDA-MB-435 cells were transfected with miR-101 mimics, an MCL-1 overexpression vector or a combination. All the cells were treated with paclitaxel. Mitochondrial membrane potential (MMP, Δψm) was determined at 48 h post-transfection. Green indicates apoptosis. **C.** MDA-MB-435 cells were transfected with miR-101 mimics, an MCL-1 overexpression vector or a combination. All the cells were treated with paclitaxel. The apoptotic cells were evaluated by Annexin V-FITC and propidium iodine staining and then analyzed by FACS. **D.** MDA-MB-435 cells were transfected with miR-101 mimics, an MCL-1 overexpression vector or their combined groups. All the cells were treated with paclitaxel. Cleaved caspase-3 and cleaved PRAP protein levels were evaluated by Western blot.

MCL-1 overexpression promotes the proliferation of MDA-MB-435 cells incubated with different doses of paclitaxel. By contrast, MCL-1 knockdown suppressed cell proliferation (Figure 6A). In paclitaxel-treated MDA-MB-435 cells, miR-101 induced green fluorescence. There was still orange fluorescence after co-transfection with a MCL-1-overexpressing vector and miR-101 in MDA-MB-435 cells (Figure 6B). These results indicated that MCL-1 overexpression could attenuate the effect of miR-101 on MMP in MDA-MB-435 cells. Additionally, MCL-1 overexpression could attenuate paclitaxel or paclitaxel combined with miR-101-induced apoptosis in MDA-MB-435 cells (Figure 6C). In addition, MCL-1 overexpression also partly suppressed apoptosis-associated cleaved caspase-3 and PARP protein levels, which were increased by paclitaxel and/or miR-101 (Figure 6D).

## DISCUSSION

Emerging research has demonstrated that miRNAs play important roles in tumor development and progression, including breast cancer [7, 10, 20, 21]. While it has been reported previously that suppression of miR-101 leads to the overexpression of MCL-1 in hepatocellular carcinoma and non-small-cell lung cancer [18, 22], the relationship between MCL-1 and miR-101 and their biological relevance in breast cancer have not yet been fully determined, especially in TNBC. In this study, the expression of miR-101 was decreased and inversely correlated with MCL-1 expression in all basal-like cell lines and in the majority of TNBC tissues. The results from the luciferase reporter assays confirmed that MCL-1 represents a direct target gene of miR-101 in TNBC cells. We assessed the effect of miR-101 and MCL-1 on breast cancer growth both *in vitro* and *in vivo*. Using two TNBC cell lines, we were able to show that miR-101 suppress the expression of MCL-1, both at the transcriptional and the post-transcriptional level. miR-101 overexpression significantly suppressed proliferation *in vitro*. These effects appeared to be mediated by inhibition of the MCL-1 oncogene, which led to cell apoptosis. Furthermore, transfection of miR-101 mimics effectively suppressed the tumorigenicity of TNBC cells in a nude mouse model. Other studies have obtained similar results. Specifically, miR-101 suppressed tumor progression by targeting MCL-1 in hepatocellular carcinoma [23] and lung cancer [24].

Studies performed by Koss B [25] indicate that endogenous MCL-1 has anti-apoptotic activity that promotes survival during BCR-ABL transformation in established BCR-ABL(+) leukemia. MCL-1 mediates the inhibition of osteosarcoma pathogenesis by miR-133a [26]. MCL-1 is overexpressed in human glioblastoma, which confers a survival advantage to tumor cells. Inhibition of MCL-1 potentiates temozolomide-induced apoptosis in gliomas [27]. In our study, we were able to show that the suppression of MCL-1 significantly inhibits proliferation and induces apoptosis *in vitro*.

Apoptosis is a critical part of various biological processes, and it represents a regulated cellular suicide mechanism that is characterized by nuclear condensation, cell shrinkage, membrane blebbing and DNA fragmentation. Apoptosis is also involved in carcinogenesis. It has been reported that miRNAs play important roles in inducing apoptosis [28] and sensitizing tumor cells to chemotherapeutic agents [29]. For example, Wang and colleagues have demonstrated that miR-101 sensitizes human bladder cancer cells to gambogic acid-induced apoptosis by inhibiting EZH2 expression [30].

Although a number of effective options have been developed for the treatment of HER2-overexpressing diseases, TNBC remains a subtype that is difficult to treat. Taxanes are considered to be some of the most active classes of compounds that work against breast cancer [31], but a major obstacle remains for successful cancer treatment. Paclitaxel inhibits proliferation and induces apoptosis in a variety of cancers. Shi *et al*. demonstrated that a PARP inhibitor reduces proliferation and increases apoptosis in breast cancer cells[32]. Additional data has shown that paclitaxel can induce apoptosis in human MDA-MB-231 breast cancer cells in a caspase-dependent manner. In our study, we were able to show that miR-101-mediated MCL-1 silencing sensitized TNBC cells to paclitaxel-induced apoptosis, whereas antagomir-mediated downregulation of endogenous miR-101 reversed the apoptotic effect. Future studies are needed to further elucidate the signaling pathways that control miR-101-mediated apoptosis in TNBC. In this study, we demonstrated that the overexpression of miR-101 enhanced the expression of apoptosis-related cleaved caspase 3 and PARP by targeting MCL-1 and that miR-101 could enhance the expression of paclitaxel-induced cleaved caspase 3 and PARP in TNBC cells. These results show that miR-101 transfection increases chemotherapeutic drug-induced apoptosis in TNBC.

Our data suggest that the tumor suppressor miR-101 represses TNBC progression and sensitizes TNBC cells to paclitaxel treatment by directly targeting MCL-1. Our findings provide significant insights into the molecular mechanisms underlying breast carcinogenesis and may have relevance for the development of novel, targeted therapies for TNBC.

## MATERIALS AND METHODS

### Cell lines and transfection

The following cell lines were obtained from the American Type Culture Collection (Manassas, VA, USA) and were passaged in our laboratory for less than six months after the frozen aliquots were thawed: five breast cancer cell lines with a luminal transcriptional profile (BT-483, T47D, MCF-7, SKBR3 and BT-474), three breast cancer cell lines with a basal-like transcriptional profile (MDA-MB-435, MDA-MB-231, and MDA-MB-468) and a normal mammary epithelial cell line (MCF-10A). Basal-like breast cancer is also known as triple-negative breast cancer (TNBC) [33]. All cells were maintained according to the supplier's instructions. Before use, all cell lines were authenticated with short tandem repeat DNA profiling and were found to be free of mycoplasma infection. Plasmids, miRNAs, and small interfering RNAs (siRNAs) were transfected into the cells at the indicated concentrations using Lipofectamine 2000 (Invitrogen, Carlsbad, CA, USA) according to the manufacturer's instructions.

### Clinical samples

Tissue samples from 22 cases of TNBC and the corresponding paired normal adjacent tissues, collected from September 2010 to May 2011 (Table 1), were analyzed using quantitative real-time PCR (qRT-PCR). Resected cancerous tissues and paired normal mammary tissues were immediately cut into pieces and stored in RNAlater (Ambion). The tissue microarrays consisted of 86 TNBC tissues (including the 22 collected samples). Histopathology was used to confirm TNBC in the samples collected from September 2010 to September 2012. The matched paracarcinoma tissues were used as the control samples. The specimens were stored in the Department of Specimens and Resources at Sun Yat-Sen University Cancer Center. The specimens were obtained during surgery and were formalin fixed and embedded in paraffin using standard methods. Immunohistochemistry of ER, PR and HER2 expression was performed in the Pathology Department of Sun Yat-Sen University Cancer Center. None of the patients included in the present study had received any chemotherapy or radiation therapy prior to the study, and their complete clinical data were available and reviewed, including age, histologic type, lymph node status, tumor size, stage, local relapse, distant metastatic relapse, ER status, PR status, and HER2 status. Histologic type was based on the TNM staging system, and the types were reclassified according to WHO classification and tumor stage (American Joint Committee on Cancer classification). Patient follow-up included a review of their records and telephone calls. The patients were grouped according to age, lymph node status, tumor size and stage. This study was approved by the Ethics Committee of Sun Yat-Sen University Cancer Center Health Authority. Written informed consent for participation in the study was obtained from all participants. The procedures for collecting and using tissues from the patients were in accordance with the ethical standards established in the Declaration of Helsinki.

### qRT-PCR

Total RNA was extracted from cells with TRIzol reagent (Invitrogen, Carlsbad, USA). Reverse transcription and qRT-PCR reactions were performed with a SYBR-green-containing PCR kit (Qiagen, Germantown, USA). The fold change was determined as 2^−ΔΔCt^. The Ct is the fractional cycle number at which the fluorescence of each sample passes the fixed threshold. ΔCt was calculated by subtracting the Ct of snRNA U6 from the Ct of the miRNA of interest. ΔΔCt was calculated by subtracting the ΔCt of the reference sample (paired non-tumorous tissue for the surgical samples) from the ΔCt of each sample. The primers for qRT-PCR detection of MCL-1 mRNA (F: TAAGGACAAAACGGGACTGG; R: CCTCTTGCCACTTGCTTTTC) were synthesized by Invitrogen. All qRT-PCR was performed with a Bio-Rad C1000 Multicolor Real-Time PCR Detection System (USA).

### *In situ* hybridization (ISH) analysis

*In situ* hybridization procedures were carried out as previously described. miR-101 miRCURYTM LNA custom detection probes (Exiqon, Vedbaek, Denmark) were used for ISH. The 5′-3′ sequence (enhanced with LNA) was UACAGUACUGUGAUAACUGAA with digoxigenin (DIG) at the 5′ and 3′ ends. Hybridization, washing, and scanning were performed according to the manufacturer's instructions. Staining intensity was scored as 0 (negative), 1+ (weak), 2+ (medium) or 3+ (strong). Low expression was defined as a staining intensity of 0, 1, 2 or 3 with < 10% of cells being stained or an intensity of 0 or 1 with < 50% of cells stained. High expression was defined as an intensity of 2 or 3 with < 10% of cells stained or an intensity of 1, 2 or 3 with < 50% of cells stained.

### Vector construct

An MCL-1-expressing vector was constructed. Full-length MCL-1 cDNA was purchased from GeneCopoeia TM (USA) and was subcloned into the pcDNA3.1(+) vector. The 3′UTR of MCL-1 (pMIR-MCL-1) or the 3′UTR with mutated binding sites for miR-101 (pMIR-MCL-1-mut) was synthesized by Invitrogen (China) and inserted into pMIR-REPORT, which expresses the firefly luciferase plasmid (Promega).

### Dual-Luciferase reporter assay and 3′UTR binding site mutagenesis

MDA-MB-435 cells (6×10^4^) were seeded in 24-well plates immediately prior to transfection. The pMIR-Mcl1 and pMIR-Mcl1-mut constructs were transfected into MDA-MB-435 cells using Lipofectamine 2000 (Invitrogen) according to the manufacturer's instructions. The miR-101 mimic was co-transfected where indicated. Forty-eight hours post-transfection, the cells were assayed for both firefly and renilla luciferase with the dual luciferase glow assay (Promega). Transfection experiments were performed in duplicate, and each experiment was repeated at least three times.

### Cell proliferation assay and cell apoptosis analysis

Cell proliferation at 48 h was determined using the cell proliferation reagent MTT (Roche Applied Science, Mannheim, Germany) according to the manufacturer's protocol. For cell apoptosis analysis, the cells were transfected as described in the previous section, and the apoptotic rate was detected with Annexin V/FITC staining and flow cytometry (FACSCalibur flow cytometer; BD Biosciences, Franklin Lakes, NJ, USA).

### Detection of mitochondrial membrane potential (MMP, Δψm) using JC-1

To measure the mitochondrial membrane potential (Δψm), 5,5′,6,6′-tetrachloro-1,1′,3,3′-tetraethylbenzimidazolylcarbocyanine iodide (JC-1), which is a sensitive fluorescent probe kit for Δψm, was used on the cells (Biyuntian Biochemistry Limited Company, China). Treated or untreated cells were cultured in 24-well plates for 24 h, washed with PBS and incubated with a working solution of JC-1 for 20 min at 37°C. Then, the cells were rinsed twice with PBS, stained with 1 mL of 10% DMEM medium containing 5 μmol/L JC-1, resuspended in 1 mL of ice-cold PBS, washed with PBS and resuspended in 500 μL of PBS. The stained cells were analyzed using a fluorescence microscope to determine color changes in the florescence from red to green.

### Immunohistochemical staining

Immunohistochemical staining was performed using an UltraSensitive S-P Kit (Maixin Biotechnology Company, Fuzhou, China). The color was developed using DAB as the chromogen. Then, the slides were counterstained with Mayer's hematoxylin and mounted for evaluation under a microscope (OLYMPUS BX-51, Osaka, Japan). Two independent pathologists who were blinded to the clinicopathological information scored the samples. Staining was scored for intensity (1, 0+; 2, 1+; 3, 2+; 4, 3+) as well as the percentage of membranous and cytoplasmic staining in malignant cells (1, 0-25%; 2, 26-50%; 3, 51-75%; 4, 76-100%). The final score was obtained by multiplying the intensity score by the score for the percentage counts. A score of < 8 was considered to be low expression, and a score of > 8 was considered to be high expression.

### Western blot

Sodium dodecyl sulfate-polyacrylamide gel electrophoresis (SDS-PAGE) was performed on protein lysates from the cells, and the target proteins were detected with primary antibodies recognizing MCL-1 (Santa Cruz, USA), Caspase-3, Cleaved PARP and GAPDH (Cell Signaling). After incubation with the appropriate horseradish peroxidase (HRP)-conjugated secondary antibodies (Jackson ImmunoResearch), the protein bands were visualized using enhanced chemiluminescence (ECL) Western blot detection reagents and then analyzed with the Bio Image Intelligent Quantifier 1-D (Version 2.2.1, Nicon-BioImage Ltd., Japan).

### Human tumor xenograft model

To investigate the antitumor effects of miR-101 *in vivo*, female BALB/c-nude mice (4–6 weeks old; Vital River Laboratories Animal, Beijing, China) were injected subcutaneously in the right fourth mammary gland with 5×10^6^ MDA-MB-435-Luc cells in 100 μL of PBS with a 30-gauge needle. Tumor formation was monitored by palpation, and tumor size was measured twice per week using calipers. Tumor volume was calculated using the standard formula: tumor volume = (width^2^×length×π)/6. When the tumors reached ∼50 mm^3^, the mice were noninvasively imaged using the IVIS *In Vivo* Imaging System (Xenogen, Alameda, CA) to confirm tumor growth. Then, the mice were randomly assigned to one of four groups (*n* = 10 mice per group): miR-LNA, miR-101-LNA, miR-scramble or miR-101 mimics. All groups received intratumoral injections twice a week for three consecutive weeks. Mouse weight and tumor progression were monitored daily using the IVIS system with Living Imaging software (Xenogen). On day 32, the mice were euthanized by spinal dislocation, and the tumors and hearts were immediately harvested, weighed, and analyzed. All experiments were performed in accordance with institutional guidelines and were approved by the animal care and use committee at the University of Sun Yat-Sen University Cancer Center.

### Statistical analysis

All values are expressed as the mean ± standard error of the mean. All experiments using cell lines were repeated a minimum of 3 times. Statistical significance was reported if the *p*-value was < 0.05 using an unpaired Student's *t*-test.
